# Functional Divergence of Poplar Histidine-Aspartate Kinase HK1 Paralogs in Response to Osmotic Stress

**DOI:** 10.3390/ijms17122061

**Published:** 2016-12-08

**Authors:** François Héricourt, Françoise Chefdor, Inès Djeghdir, Mélanie Larcher, Florent Lafontaine, Vincent Courdavault, Daniel Auguin, Franck Coste, Christiane Depierreux, Mirai Tanigawa, Tatsuya Maeda, Gaëlle Glévarec, Sabine Carpin

**Affiliations:** 1 Laboratoire de Biologie des Ligneux et des Grandes Cultures (LBLGC), Université d’Orléans, INRA, USC1328, 45067 Orléans, France; francois.hericourt@univ-orleans.fr (F.H.); francoise.chefdor@univ-orleans.fr (F.C.); ines.djeghdir@univ-orleans.fr (I.D.); melanie.larcher@univ-orleans.fr (M.L.); daniel.auguin@univ-orleans.fr (D.A.); christiane.depierreux@univ-orleans.fr (C.D.); 2 Biomolécules et Biotechnologies Végétales (BBV), EA 2106, Université François Rabelais de Tours, 31 avenue Monge, 37200 Tours, France; fllafontaine@univ-tours.fr (F.L.); vincent.courdavault@univ-tours.fr (V.C.); gaelle.glevarec@univ-tours.fr (G.G.); 3 Centre de Biophysique Moléculaire (CBM), CNRS, 45071 Orléans, France; franck.coste@cnrs-orleans.fr; 4 Institute of Molecular and Cellular Biosciences, The University of Tokyo, 1-1-1 Yayoi, Bunkyo-ku, Tokyo 113-0032, Japan; las17mirai@yahoo.co.jp (M.T.); maeda@iam.u-tokyo.ac.jp (T.M.)

**Keywords:** histidine-aspartate kinase (HK), histidine-containing phosphotransfer protein (HPt), multistep phosphorelay (MSP), osmosensing, *Populus*, drought signaling

## Abstract

Previous works have shown the existence of protein partnerships belonging to a MultiStep Phosphorelay (MSP) in *Populus* putatively involved in osmosensing. This study is focused on the identification of a histidine-aspartate kinase, HK1b, paralog of HK1a. The characterization of HK1b showed its ability to homo- and hetero-dimerize and to interact with a few Histidine-containing Phosphotransfer (HPt) proteins, suggesting a preferential partnership in poplar MSP linked to drought perception. Furthermore, determinants for interaction specificity between HK1a/1b and HPts were studied by mutagenesis analysis, identifying amino acids involved in this specificity. The *HK1b* expression analysis in different poplar organs revealed its co-expression with three *HPts*, reinforcing the hypothesis of partnership participation in the MSP in planta. Moreover, HK1b was shown to act as an osmosensor with kinase activity in a functional complementation assay of an osmosensor deficient yeast strain. These results revealed that HK1b showed a different behaviour for canonical phosphorylation of histidine and aspartate residues. These phosphorylation modularities of canonical amino acids could explain the improved osmosensor performances observed in yeast. As conserved duplicates reflect the selective pressures imposed by the environmental requirements on the species, our results emphasize the importance of *HK1* gene duplication in poplar adaptation to drought stress.

## 1. Introduction

Water deficit is one of the most important environmental stresses affecting plant growth. At the molecular level, many genes are repressed or induced to face this constraint [1] but the signaling pathways by which plants regulate gene expression to activate adaptive responses is still unclear. Understanding the sensing mechanism of drought stress and signal transduction is thus a challenge to develop new stress tolerant plants. In *Saccharomyces cerevisiae*, increasing extracellular osmolarity induces the activation of the High-Osmolarity Glycerol (HOG) Mitogen-Activated Protein (MAP) kinase pathway leading to osmotic responses [2,3]. This HOG pathway is controlled by two upstream osmosensing branches—Sln1p and Sho1p branches—wherein a classical MultiStep Phosphorelay (MSP) system ruled by a Sln1p receptor is acting [4,5]. This MSP is composed by Sln1p; a hybrid-type histidine-aspartate kinase (HK) osmosensor; a histidine-containing phosphotransfer (HPt) protein Ypd1p; and the response regulator (RR) protein Ssk1p. Under nonosmotic stress conditions, Sln1p, in a dimerized form [6], phosphorylates its own histidine kinase domain and transfers the phosphate group to its own C-terminal receiver domain (RD) [2,3]. This phosphate group is then transferred to Ypd1p and finally to Ssk1p, leading to the inactivation of the HOG pathway. On the other hand, under hyper-osmotic stress conditions, Sln1p phosphorylation is repressed leading to the activation of the HOG MAP kinase cascade [2,3,7]. Activated Hog1p MAP kinase is essential for glycerol accumulation, re-establishing cellular turgor pressure to allow the survival of yeast cells under hyper-osmotic stress conditions [8]. However, a constitutive phosphorylation of Hog1p due to *sln1* deletion under normal osmolarity conditions leads to cell death [2]. This lethality is suppressed by overexpression of the phosphotyrosine phosphatase Ptp2p which dephosphorylates Hog1p [9,10].

In bacteria, osmosensing is performed by the so-called two-component system from which MSP has evolved. The membrane sensor kinase EnvZ, and the cytoplasmic response regulator OmpR, control bacteria responses to osmotic stress. It is now well established that osmotic imbalance directly activates EnvZ activity by inducing a more folded conformation of its cytoplasmic part [11,12]. In yeast, even though Sln1p has a similar HK domain organization to the bacterial osmosensor EnvZ [6,13], there is no evidence of a direct effect of osmotic imbalance on Sln1p structure. On the other hand, Sln1p (i) has a kinase activity enhanced by increased glycerol intracellular concentration [14]; (ii) responds to changes in turgor pressure [15]; (iii) is able to respond to changes induced by osmolarity in the cell wall [16] and (iv) its dimerization is disrupted by the osmotic stress leading to the inactivation of the kinase [17]. Finally, the importance of Sln1p in osmosensing has been extensively demonstrated even if, conversely to EnvZ, the structural mechanism of the kinase activation is not clearly established.

In *Arabidopsis thaliana*, a MSP seems also to participate to drought perception. The *Arabidopsis* hybrid-type histidine-aspartate kinase AHK1, a plasma membrane protein, is able to perceive the osmotic stress in yeast and activate the HOG pathway leading to the proposal of AHK1 as an osmosensor [18,19]. In plants, analysis of *ahk1* mutants gave evidence that AHK1 (i) acts as a positive regulator of osmotic stress signaling; (ii) is involved in drought tolerance [19,20] and (iii) controls plant water status probably by determining stomatal density [21,22]. The *Arabidopsis* genome encodes five authentic HPts (AHP1 to AHP5) [23] and it has been demonstrated that AHK1 can interact with only AHP2 and that the RD alone is sufficient for this interaction [24]. The *Arabidopsis* genome also encodes 24 RRs [25] and analysis of *arr18* and *ahk1* mutants, presenting the same germination phenotype, led to the conclusion that ARR18 functions downstream of AHK1 [26]. Moreover, the observed interaction between ARR18 and AHP2 [27] supports the idea that downstream partners in AHK1 cascade could be at least AHP2 and ARR18 and constitute a MSP involved in *Arabidopsis* osmosensing [28]. However, the complete composition of the MSP and the osmotic imbalance effect on AHK1 activity to induce plant responses remains to be determined.

In *Populus*, HK1, a protein homologous to AHK1 [29], ten cDNAs encoding HPts (HPt1 to HPt10) [30] and nine cDNAS encoding RRs (RR12–RR16, RR18–RR19, RR21–RR22) [31] have been isolated. Characterization of corresponding proteins led to the identification of a preferential partnership which could constitute a MSP putatively involved in *Populus* drought sensing [29,30,31,32,33].

After a new in silico *Populus* genome screening for HK1, we found a HK1 paralog. In the present work, we have isolated this second *HK1* gene and we present the characterization of this new protein (HK1b) in comparison with the former one (HK1a). We demonstrated that HK1b is able to form homodimers as well as heterodimers with HK1a in a yeast two-hybrid system. We also showed the preferential partnership of HK1b with few HPts and the identification of amino acids potentially determinant for both HK1/HPts interaction specificity in a mutagenesis study. This study highlights a slight divergence of partnership between both receptors. Moreover, mRNAs expression analysis revealed a co-expression of *HK1b* and *HK1a* with their respective partners in different poplar organs. Finally, we showed that HK1b can act as Sln1p in an osmosensing pathway in an osmodeficient yeast strain by a functional complementation assay, as it was shown for HK1a, but with a better efficiency. Furthermore, mutagenesis analysis of HK1b allowed us to pinpoint a non-classical phosphorylation site and functional divergence for these two paralogs. All these data gave us some new evidence that genes duplication can lead to functional divergence and that *MSP* genes duplication could be important for plant adaptation.

## 2. Results

### 2.1. Identification of Histidine-Aspartate Kinase 1 (HK1) Gene Duplication in Populus

A BLAST analysis on the latest version of the *Populus trichocarpa* genome database (v3.0) in Phytozome, allowed us to identify and isolate a second homologous gene of *Arabidopsis thaliana AHK1*. This gene presents 91.27% identity with the *HK1* poplar gene previously characterized [19] and corresponds to a paralogous gene in the *Populus* genome. This new *HK1* gene is located on chromosome 5 while the former *HK1* gene is on chromosome 7. This second *HK1* gene was named *HK1b* and, accordingly, the previously isolated *HK1* gene was renamed *HK1a*.

The deduced amino acid sequence of the *HK1b* gene shows a typical protein topology of a histidine-aspartate kinase with two transmembrane domains: a transmitter domain and a receiver domain. This protein shares 96.35% of overall similarity with HK1a.

**Figure 1:**
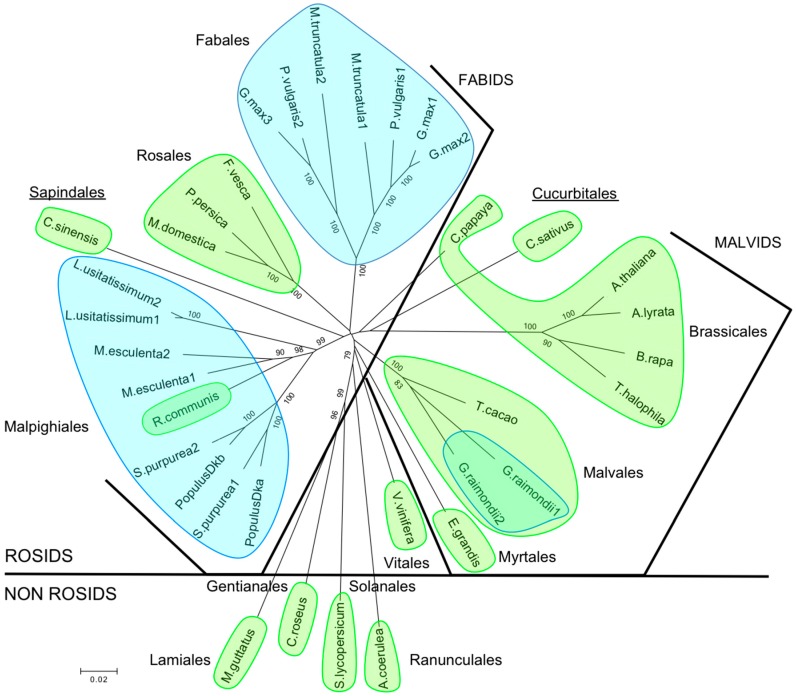
Unrooted relationship tree of Histidine-aspartate Kinases 1 (HK1s) from *Populus* and other plants. Amino acid sequences of HK1a (*Populus* Dka, accession No. AJ937747) and HK1b (*Populus* Dkb, accession No. LT622839) were aligned with HK1 homologous proteins of *Arabidopsis* (*A. thaliana*, AT2G17820 and *A. lyrata*, 480680), saltwater cress (*T. halophila*, XM_006409179), turnip (*B. rapa*, Brara.G00235), cucumber (*C. sativus*, Cucsa.322710), papaya (*C. papaya*, evm.TU.supercontig_6.277), cocoa (*T. cacao*, Thecc1EG000177), cotton (*G. raimondii*, Gorai.007G049200 and Gorai.003G008400), peach (*P. persica*, Prupe.7G170700), apple (*M. domestica*, MDP0000276711), strawberry (*F. vesca*, gene14583-v1.0-hybrid), sweet orange (*C. sinensis*, orange1.1g001044m.g), rose gum (*E. grandis*, Eucgr.I02335), grape vine (*V. vinifera*, GSVIVG01018749001), Colorado blue columbine (*A. caerulea*, Aqcoe6G223000), tomato (*S. lycopersicum*, Solyc02g083680), Madagascar periwinkle (*C. roseus*, AF534893), yellow monkeyflower (*M. guttatus*, Migut.L00691), purple willow (*S. purpurea*, SapurV1A.0698s0020 and SapurV1A.0130s0330), castorbean (*R. communis*, 29656.t000014), cassava (*M. esculenta*, Manes.02G106100 and Manes.01G147600), flax (*L. usitatissimum*, Lus10041891.g and Lus10028438.g), barrelclover (*M. truncatula*, Medtr5g022470 and Medtr8g075340), common bean (*P. vulgaris*, Phvul.002G107100 and Phvul.003G264600) and soybean (*G. max*, Glyma01g36950, Glyma11g08310 and Glyma02g05220) by CLUSTALW and were represented as a phylogram constructed with the neighbour-joining method and 1000 bootstrapping replicates. The bootstrap values higher than 75% are indicated on the tree. The scale bar represents 0.02 amino acid substitution per site. Each classification order is circled and classification clades are separated by thick black lines. The two underlined orders belong to the opposite clade. Plant species with one HK1-like gene are highlighted in green and plant species with at least two *HK1-like* genes are highlighted in blue.

The relationship of this pair of poplar HK1 with other HK1 homologous proteins from different plants is represented as an unrooted tree (Figure 1). Branch lengths are proportional to sequence divergences, thus illustrating similarities between HK1 sequences. It appears that our poplar HK1a/1b group with species also containing two copies of HK1-like protein in the Malpighiales order, excepting *Ricinus communis*. Duplications seem to be a common feature in the Fabids clade since species in Fabales order also present at least two copies of HK1-like protein. On the opposite side, species in the Malvids clade possess only one copy of HK1-like protein, with the exception of *Gossypium raimondii*. It is noteworthy that, according to the HK1-like sequence, two species do not gather in the clade to which they belong: *Citrus sinensis* belongs to Malvids clade and *Cucumis sativus* belongs to Fabids clade. These results suggest that some plant species have evolved toward a duplication of their *HK1* gene and constitute a feature shared by numerous plant species in the Fabids clade.

### 2.2. HK1 Paralogs Are Able to Homo- and Heterodimerize

**Figure 2:**
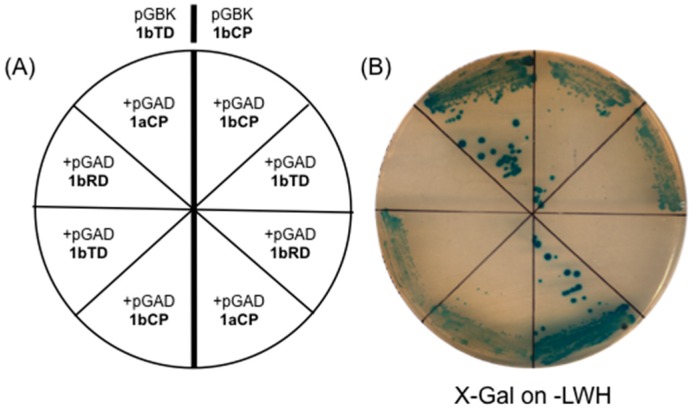
Homodimerization and heterodimerization of HK1b. (**A**) Two-hybrid interaction tests: HK1b-CP and HK1b-TD were tested with different domains of HK1b and HK1a as indicated (CP: cytoplasmic part, TD: transmitter domain, RD: receiver domain); (**B**) Transformed yeasts were streaked onto selective medium (-LWH) for the growth test and X-Gal test.

To test the ability of HK1b to self-interact, as shown for HK1a, we conducted a two-hybrid (2H) assay with the cytoplasmic part of HK1b (HK1b-CP) expressed from both 2H vectors in the LexA based 2H system. The results did not indicate any interaction, even when the transmitter domain (TD) was used, although it was demonstrated that TDs are the minimal domains for homodimerization. We suspected a conformational problem with the fusion protein used in this 2H system. Therefore, we changed this system for a Gal4 based system. In this new system, we tested all the possibilities of self-interaction of HK1b (Figure 2A) and we were able to see homodimerization of this protein (Figure 2B). As it was observed in a former study on HK1a, HK1b is able to interact with itself and this interaction is mediated by TD, since no interaction was observed when solely RD is tested.

Furthermore, we tested the ability of HK1b to interact with HK1a for a possible heterodimerization. In this 2H system, HK1b and HK1a can interact and TD alone is sufficient to support this interaction (Figure 2B).

### 2.3. HK1 Paralogs Do Not Share All HPt Partners

A global yeast 2H analysis was conducted to study interactions between HK1b and HPts. Therefore, HK1b was tested in the bait configuration by expressing its receiver domain (HK1b-RD) since RD alone presents a stronger interaction with HPts than the complete cytoplasmic part [30].

**Figure 3:**
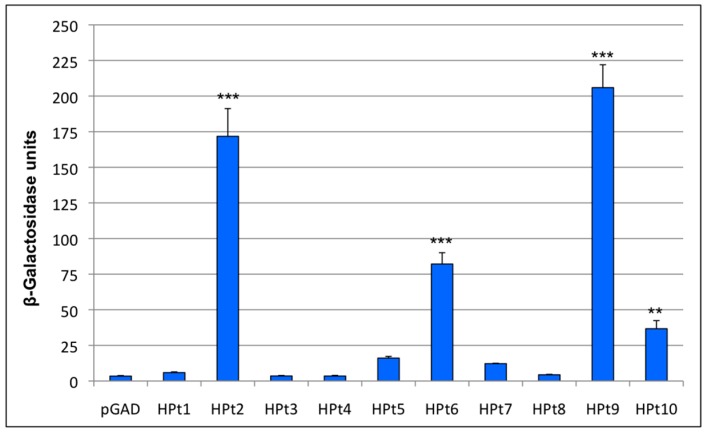
Two-hybrid quantitative tests. β-Galactosidase activity for each HK1b-RD/HPt interaction is plotted with mean values of at least four independent assays performed in triplicate and represented with standard error bars. The interaction background control was measured with an empty prey vector (pGAD) and significant differences from this control are represented by ** with *p* < 0.01 or *** with *p* < 0.001.

This interaction analysis revealed reporter genes activation on interaction-selective medium for five HPts among the 10 tested, although at different levels (Figure S1A) with homogenous levels of expression (Figure S1B). To refine those results, we quantified the β-Galactosidase activity for each pair of HK1b/HPt (Figure 3) since this reporter gene activity reflects the strength of the detected interactions. The pLex-HK1b-RD construct with the empty pGAD vector was used as an interaction background control. These quantifications showed that HPt1, 3, 4, 5, 7, 8 presented an activity level similar to the control. Conversely, HPt2, 6, 9, 10 interacted in a significant way with HK1b-RD and among them, HPt9 and 2 presented the strongest β-Galactosidase activity. These results are in agreement with those obtained from qualitative assays (Figure S1) and are partially overlapping to those obtained from HK1a/HPts interaction analysis. Indeed, HK1a was shown to interact with HPt2, 6, 7, 9 and 10 where HPt2 and 9 corresponded to the strongest interacting partners [30]. These results suggest that HK1a and HK1b share the main HPt partners, HPt2 and HPt9, but not all of them, as observed with HPt7.

### 2.4. Amino Acids Involved in the Specificity of HK1/HPts Interactions

**Figure 4:**
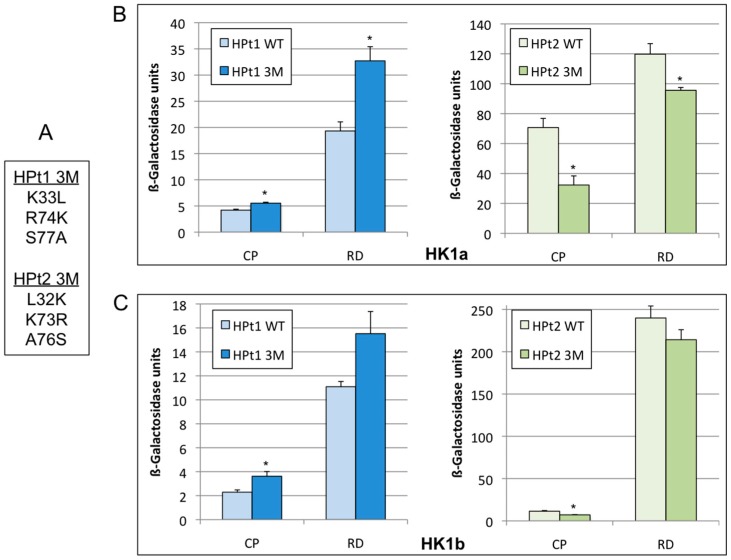
Interaction test with HPt mutants. (**A**) Mutated amino acids in the HPt1 and HPt2 triple mutants (3M); (**B**) β-Galactosidase activity for each HK1a/HPt interaction is plotted with mean values of at least four independent assays performed in triplicate and represented with standard error bars. Significant differences from the wild-type (WT) interaction are represented by * with *p* < 0.05; (**C**) Same dosage for each HK1b/HPt interaction is plotted in the same representation. Significant differences from the wild-type (WT) interaction are represented by * with *p* < 0.05.

In order to understand how these interactions mediate their specificity, we decided to determine the residues involved in this specificity. To select potential amino acids, we performed an alignment of all HPts with each other according to their ability to interact or not with HK1a/b (Figure S2). This alignment allowed us to pinpoint few amino acid candidates according to their presence in the strong-interacting HPt group and absence in the non- or weak-interacting HPt group. Moreover, we used the solved structure of the *Arabidopsis* AHK5_RD_/AHP1 complex [34] to focus our choice on the HPt helices known to interface with the RD (Figure S2). In this close relative complex, helices α2, α3 and α4 of AHP1 formed a groove where helix α1 from AHK5_RD_ can insert. We selected three of them for a mutagenesis experiment, even though residue in mutation site 2 is partially present in both groups. Indeed, the lysine residue is also present in HPt4 and HPt8 but a proline residue is present 4–5 amino acids upstream which may induce a different structure and present in HPt10, but this HPt is able to interact, although weakly. HPt1 and HPt2 were used as representatives of both groups, to generate triple mutants and test them in interaction with HK1a and HK1b. The mutations introduced in HPt1 (K33L/R74K/S77A) correspond to amino acids present in HPt2 (L32K/K73R/A76S) at the homologous position and vice versa (Figure 4A). Consequently, a yeast 2H analysis was conducted with HK1a/b, tested in bait configuration (complete cytoplasmic part, HK1-CP or receiver domain only, HK1-RD) and HPts in prey configuration.

This interaction analysis revealed that HPt1 triple mutant (3M) allows better growth and blue colouring with HK1a (-RD or -CP) compared to wild type HPt1 (Figure S3A). On the other hand, HPt2 triple mutant decreases growth and colouring in comparison to wild type HPt2. As previously reported, HK1a-RD/HPts interactions are stronger than HK1a-CP/HPts interactions. Proteins expression in each interacting yeast clone was checked and showed a comparable level of protein expression (Figure S3B). The same results were obtained when HK1b-RD and HK1b-CP were used (data not shown).

To refine those results, the β-Galactosidase activity for each pair of HK1/HPt (Figure 4) was measured to estimate the strength of these interactions. As an interaction control, HK1-CP or HK1-RD with the wild-type HPt1 or HPt2 were used. These quantifications showed that HK1a-CP or HK1a-RD/HPt1 triple mutant (3M) interactions have a significantly higher level of β-Galactosidase activity compared to the wild-type (Figure 4B). Conversely, HK1a-CP or HK1a-RD/HPt2 triple mutant (3M) interactions have a significantly lower level of β-Galactosidase activity compared to the wild-type. These results are in agreement with those obtained from qualitative assays (Figure S3A) and indicate that HPt1 3M is able to achieve a better interaction than wild-type protein and HPt2 3M is able to reduce the interaction. The same results were obtained with HK1b although the increase and decrease observed were only significant for HK1b-CP/HPts interaction (Figure 4C). These data are indicative of the involvement of these residues in the specificity of interaction between both HK1 proteins and HPts but are also indicative that others amino acids must participate in this specificity.

### 2.5. In Planta Validation of HK1b/HPts Interactions

**Figure 5:**
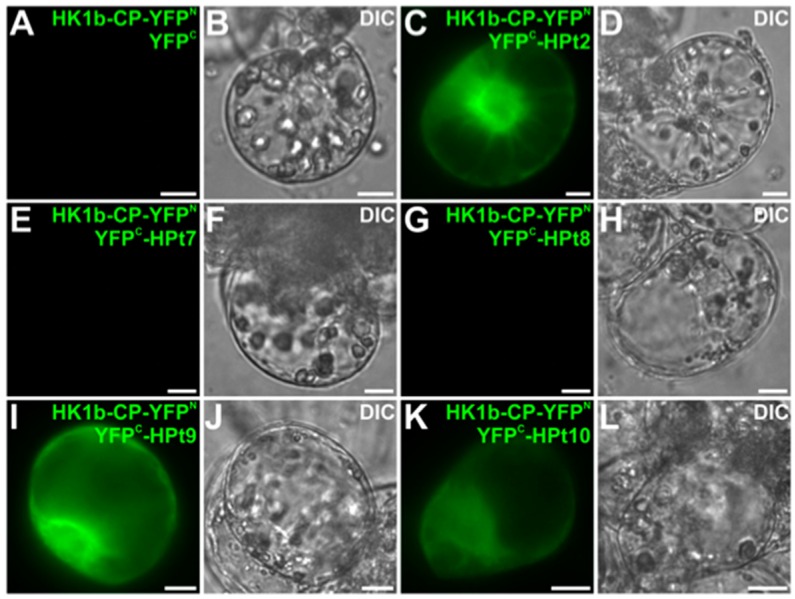
Analysis of HK1b-CP/HPts interactions in Bimolecular Fluorescent Complementation (BiFC) assays. Cells were co-transformed using a combination of plasmids expressing HK1b-CP/HPt2 (**C**,**D**); HK1b-CP/HPt7 (**E**,**F**); HK1b-CP/HPt8 (**G**,**H**); HK1b-CP/HPt9 (**I**,**J**) and HK1b-CP/HPt10 (**K**,**L**) as indicated. As a negative control, the combination of plasmids expressing HK1b-CP/YFP^C^ (**A**,**B**) was used. The morphology was observed by differential interference contrast (DIC) microscopy. Scale bar = 10 µm.

To confirm in planta the HK1b/HPts interactions observed in yeast, we tested whether HK1b can interact with HPts using Bimolecular Fluorescent Complementation (BiFC) assays in *Catharanthus roseus* cells. For such analysis, we tested HPt2, HPt9 and HPt10 as interacting partners in the 2H system. HPt6 was not tested since its co-expression with HK1b was not detected in planta [30]. Furthermore, BiFC assays were also conducted for HK1b interactions with HPt7 and HPt8 to confirm the negative results obtained in 2H tests. HPt coding sequences were fused to the C-terminal (YFP^C^) fragments of yellow fluorescent protein (YFP) at its C-terminal end to produce YFP^C^-HPt fusion proteins while the HK1b-CP coding sequence was fused to the N-terminal (YFP^N^) fragments of YFP at its N-terminal end producing HK1bCP-YFP^N^. After co-transformation, the different combinations of HK1b-CP and HPt constructs led to the formation of a BiFC complex within plant cells only for HPt2 (Figure 5C), HPt9 (Figure 5I) and HPt10 (Figure 5K). By contrast, no YFP reconstitution could be visualized when co-expressing HK1b-CP with HPt7 (Figure 5E) and HPt8 (Figure 5G), nor with YFP^C^ fragment alone (Figure 5A), thereby validating the specificity of interaction between HK1b-CP and HPt2, 9 and 10 in planta. This interaction study confirmed in planta the interactions detected in yeast using 2H analysis and also the lack of interaction between HK1b and HPt7.

### 2.6. In Planta Expression Profile of HK1b

**Figure 6:**
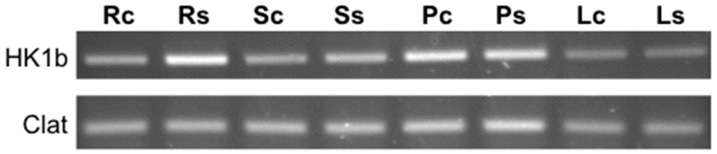
Gene expression of *HK1b*. *HK1b* cDNAs were amplified in roots (R), stems (S), petioles (P) and leaf blades (L) in control (c) and stress (s) conditions (PEG 50 g/L). The *Clathrine* (Clat) gene was used as control.

A former expression analysis performed on *HK1a* and *HPts* showed a co-expression of this *HK* and three *HPt* genes in planta [30]. Therefore, we analysed the expression pattern of *HK1b* in the same conditions where expression was monitored in four organs by RT-PCR in control and hyperosmotic (PEG 50 g/L) conditions (Figure 6). *Clathrin* was used as the amplification control gene. Results showed that *HK1b* presents a constitutive expression since it was detected in all organs tested and in both conditions. Expression seems to be more important in petioles and stressed roots than in leaf blades. A slight increase of *HK1b* mRNA level seems to be observable in stressed roots as shown for *HK1a* [29]. This expression profile is highly similar to the one observed for *HK1a* and showed that both poplar *HK1* genes are co-expressed in all studied organs as well as *HPt2*, *HPt7* and *HPt9*, except for *HPt10*, the weakest significantly interacting HPt partner, for which the co-expression pattern was restricted to the leaf blades [20].

### 2.7. Functional Divergence between Both HK1s

**Figure 7:**
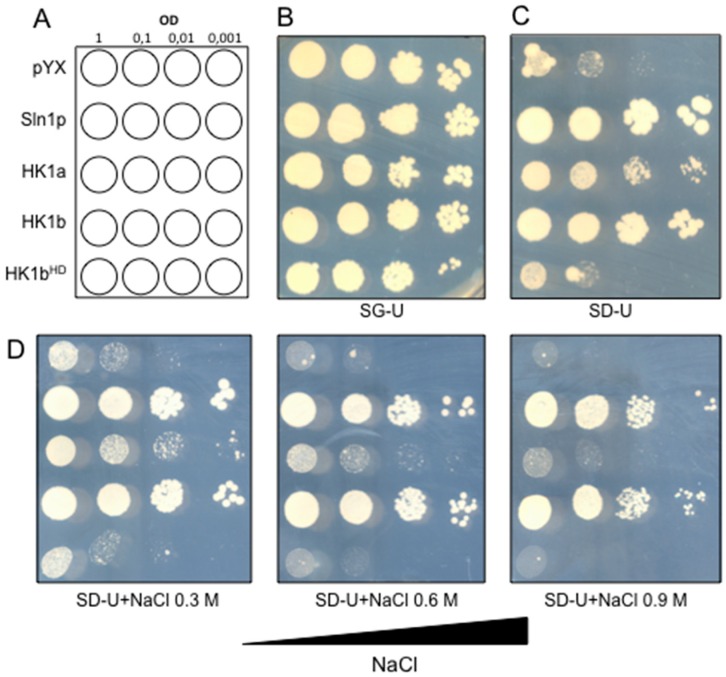
Functional complementation assay. (**A**) Different constructs were introduced into the osmodeficient strain (MH179) as indicated and spotted onto different media at a density corresponding to an optical density (OD)_600_ = 1 and three 10-fold dilutions as indicated. The resulting transformants were spotted onto SG-Ura medium for growth control (**B**) and onto SD-Ura medium for growth rescue (**C**). Finally, transformants were spotted onto SD-Ura+NaCl media with increasing concentration of NaCl for the osmotic tolerance test (**D**).

To check the functionality of HK1b, we performed a complementation assay in a *S. cerevisiae* deletion mutant strain (MH179) where the two osmosensors are deleted (*sln1Δ sho1Δ*). This strain was transformed with plasmids carrying the wild-type *HK1b* cDNA (pYX-HK1b) or mutated *HK1b* cDNA (pYX-HK1b^HD^). The substitution in HK1a of canonical donor histidine residue or canonical receiver aspartate residue by a glutamine and an asparagine, respectively, which impairs all possibilities of phosphorylation, inhibited the complementation observed for wild-type HK1a [20]. The mutated and wild-type HK1b transformants were tested for their capacity to grow on normal or high-osmolarity medium. In parallel, the MH179 strain was transformed with a plasmid carrying the *SLN1* cDNA (pPD-Sln1) and with the empty pYX212 vector as positive and negative controls respectively, as well as *HK1a* cDNA (pYX-HK1a) for comparison (Figure 7A). In this strain, *PTP2* expression under the control of an inducible galactose promoter, allows the survival of all transformants on galactose medium (SG-Ura, Figure 7B). On glucose medium (SD-Ura), HK1b and Sln1p expressing yeasts exhibited normal growth, whereas cells transformed with the empty vector or with the vector carrying mutated *HK1b* had very limited growth (Figure 7C). This result indicated that wild-type HK1b was able to functionally replace Sln1p in yeast as observed with HK1a. On the other hand, when HK1 phosphorylation was abolished into the transmitter and receiver domain, HK1b failed to complement the *sln1Δ sho1Δ* strain (Figure 7C). These results showed that HK1b functions as a histidine-aspartate kinase under normal osmotic conditions and that this function was necessary for the complementation and recovery of the lethal phenotype of the *sln1Δ sho1Δ* yeast strain. When transformed cells were spotted on the same medium, but in presence of increasing NaCl concentration, only Sln1p and wild-type HK1b expressing yeasts were able to grow up to 0.9 M NaCl (Figure 7D). This result showed that HK1b was osmo-responsive and achieved this activity in a similar manner to the yeast osmosensor Sln1p. Moreover, the comparison between HK1a and HK1b showed a more efficient complementation for HK1b since HK1a can restore activity only up to 0.6 M NaCl (Figure 7D).

**Figure 8:**
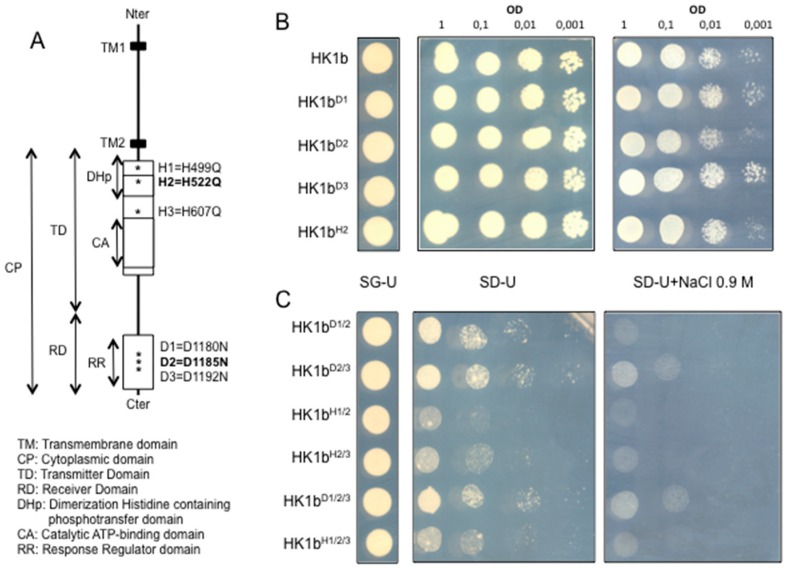
Functional complementation with HK1b mutants. (**A**) Schematic representation of HK1b with the different domains as indicated. All mutated positions are indicated with an asterisk and the corresponding mutated sequences shown on the right. The putative canonical histidine and aspartate residues are in bold; (**B**) Wild-type and single mutation constructs of HK1b were introduced into the osmodeficient strain (MH179) as indicated and spotted onto different media at a density corresponding to an OD_600_ = 1 and three 10-fold dilutions as indicated. The resulting transformants were spotted onto SG-Ura medium for growth control and onto SD-Ura medium for growth rescue. Finally, transformants were spotted onto SD-Ura+NaCl media with increasing concentrations of NaCl for the osmotic tolerance test; (**C**) Double and triple mutation constructs of HK1b were introduced into the osmodeficient strain (MH179) as indicated and spotted onto different media at a density corresponding to an OD_600_ = 1 and three 10-fold dilutions as indicated. The resulting transformants were spotted onto SG-Ura medium for the growth control and onto SD-Ura medium for growth rescue. Finally, transformants were spotted onto SD-Ura+NaCl medium with 0.9 M of NaCl for the osmotic tolerance test.

Previous works have shown that single HK1a mutations affecting canonical phosphate accepting residues (HK1a^H514^ and HK1a^D1176^) succeed in abolishing HK1a kinase activity in a functional complementation assay [30] but the same mutations in HK1b (HK1b^H522^ and HK1b^D1185^) did not (Figure 8B). The same result was obtained with single mutations affecting the two aspartate (HK1b^D1180^ and HK1b^D1192^) residues surrounding the canonical aspartate residue (Figure 8B). This fact prompted us to investigate further to understand this issue. The analysis of the HK1b sequence revealed that two other histidine (H499 and H607) or aspartate (D1180 and D1192) residues are present close to the conserved histidine (H522) or aspartate (D1185) residues putatively involved in phosphorylation (Figure 8A). We hypothesized that these residues could provide alternative phosphorylation sites. Therefore, we mutated these potential alternative residues in order to determine which one could replace the canonical residues in the phosphorylation process. We generated double and triple mutants, including canonical phosphate accepting residues, and we tested them in the functional complementation assay (Figure 8C). Although some of these mutants allowed a limited growth on SD-Ura medium, none of them were able to complement the yeast osmodeficiency when osmostic stress was applied compared to wild-type HK1b. Nonetheless, a slight difference of growth is observed for aspartate mutants compared to histidine mutants on SD-Ura medium. All these results suggested that when one phosphorylation site is missing, the two others can assume the function but when at least two phosphorylation sites are missing, the bypass mechanism is no longer possible.

## 3. Discussion

In our previous works, we isolated and characterized a *Populus* histidine-aspartate kinase, called HK1 [29,30], proposed as an osmosensor, and the first actor of a MSP committed to the poplar osmosensing pathway. Recently, a phylogenic analysis of cytokinin receptors, revealed duplication events as, for example, poplar cytokinin receptors [35]. A new analysis of the poplar genome for histidine-aspartate kinases pinpointed the existence of a *HK1* paralog. As reported by Panchy et al. [36], integrated analysis of gene families led to the observation that plant genes involved in signal transduction and stress response were mainly concerned with duplication events and were present as paralogs in the plant genome. As shown by the phylogenic tree for HK1-like proteins, firstly, we observed a clade inversion for Sapindales and Cucurbitales orders which, according to APG IV [37], belong to Malvid and Fabid clades respectively. Secondly, plant species belonging to the different clades of Rosids, Fabids and Malvids, do not share the same pattern of duplication for the *HK1* gene. Indeed, all species belonging to Malvids present only one *HK1* gene, excepting *Gossypium raimondii*. On the other hand, species belonging to Fabids have a less homogenous behavior for gene duplication since we observe two *HK1* genes in Malpighiales and Fabales orders but only one *HK1* gene in Rosales and Cucurbitales orders. As shown by the HK1 distribution in the plant kingdom, there is no link, neither between HK1 duplication and the perennial or annual plants trait, nor between HK1 duplication and the woody or herbaceous plant trait. Nevertheless, it is now established that gene duplication is an important process in plant adaptation to abiotic or biotic changing environments and that paralogs could acquire novel functions that would contribute to better plant adaptation [36]. Genes belonging to MSP systems are concerned with duplication and it is established that gene redundancy as, for example, RR genes, led to robustness in signaling systems against various perturbations and also diversification to acquire new functional or regulatory strategies over evolution [38]. According to this, we decided to characterize the new *HK1* gene, called HK1b, in order to determine if that paralog could play a different role from HK1a.

As observed for HK1a, HK1b is able to self-interact with TD, as a sufficient interacting domain, leading to the same conclusion as for HK1a, i.e., TD contains the dimerization domain [30]. This property is a common feature of HK receptors since they have to function as dimers to autophosphorylate each other in trans [17,39]. We also observed the possible heterodimerization between the two HK1 proteins, as already observed for ethylene receptors [40] or cytokinin receptors [41,42]. If the duplication of the *HK1* gene leads to a functional divergence of paralogs, the heterodimerization of HK1 could permit a greater diversity of responses to drought in plants.

To go further, we decided to investigate the HK1b partnership with the ten HPt proteins previously isolated [29,30]. As shown by the 2H assay, HK1b was able to interact significantly with four HPt proteins—HPt2, 6, 9 and 10—the main interacting partners being HPt2 and 9. These interactions were confirmed by BiFC interaction in planta. Similarly, HK1a was shown to interact mainly with HPt2 and 9, which suggests that the two poplar HK1 proteins share common HPt partners. As previously observed by RT-PCR analysis, *HPt6* expression is not detected in the different studied organs (roots, stems, petioles and leaf blades) in our experimental conditions and thus could probably not be involved in the signaling pathway in those organs. Furthermore, HPt7 is able to interact with HK1a but not with HK1b, demonstrating a discrepancy between the two HK1 proteins. This result suggests that both HK1 proteins can also have a specific partnership with HPts. As described in *Arabidopsis*, duplicates tend to have different interaction partners [43,44] and we confirmed this hypothesis for HK1 duplicates. Knowing that HPt7 is mainly expressed in roots [30], this different behavior of both HK1s towards this HPt could lead to a different involvement of these two paralogs in the drought signaling pathway in poplar roots. Therefore, both HK1 proteins seem to have a preferential partnership with some specific HPt proteins, and in order to confirm this hypothesis, we decided to identify the amino acids involved in this interaction specificity.

In this regard, knowledge on the HK/HPt complex structure is of particular interest. So far, only two examples of solved structures of HK/HPt complexes in eukaryotes are present in the literature: the complex of yeast Sln1p_RD_ with Ypd1p [45] and the *Arabidopsis* complex of AHK5_RD_/AHP1 [34]. Unlike the classical idea proposing that HPt proteins can act as functionally redundant signaling hubs, we were convinced that HPt proteins could really play a role leading to response specificity in the MSP system. Consequently, our strategy was to choose two contrasted HPt proteins—strong and weak interacting partners, HPt2 and HPt1 respectively—and by mutagenesis, interconvert critical positions in attempting to reverse their interaction profiles with both HK1 proteins. As shown in [34], the HPt interface is formed by three helices—α2, α3 and α4—and the authors proposed that some conserved residues belonging to these helices are important for interaction and also responsible for the lack of specificity. According to our hypothesis of specificity, instead of considering these conserved amino acids, we focused our efforts on specific amino acids of strong or weak interaction profiles and that are present in helices α2 to α4. This approach allowed us to pinpoint three amino acids and we showed that these amino acids could be involved in the HK1/HPt interaction specificity even though we did not recover the initial interaction level for each mutant form, supporting the idea of the involvement of other amino acids in this specificity. Otherwise, as proposed by Bauer et al. [34], additional molecular mechanisms could interfere within the HK_RD_/HPt complex formation, perhaps to provide additional specificity. Our results are in agreement with those obtained with CKI1 showing interaction specificity with only three AHP among six in *Arabidopsis* [46]. Overall, these findings strengthen the hypothesis that HPt proteins participate to a certain level of interaction specificity within the MSP system [47,48], validating HPt2 and HPt9 as partners probably involved in poplar MSP linked to drought.

In order to assign a drought receptor role to HK1b, the kinase function and the osmotic stress effect on this function needed to be validated as shown for AHK1 [18,19] and poplar HK1a [30]. Functional complementation tests were performed using a double mutant yeast strain, MH179 *sln1Δ sho1Δ*. On glucose medium, only strains transformed with functional kinases can grow and when supplemented with NaCl, triggering a hyper-osmotic stress, only strains containing functional osmosensing can survive. The results showed that HK1b is a functional kinase as Sln1p and HK1a and this was confirmed by the lethality observed for the yeast expressing HK1b^HD^, mutated on its two phosphorylatable residues (H522 and D1185), leading to an unphosphorylatable receptor and a nonfunctional kinase. As we already shown for HK1a, the function of this protein during the osmotic stress can activate the HOG pathway but a single mutation on each canonical amino acid was sufficient to abolish HK1a functioning [30]. Unexpectedly, single mutations on canonical amino acids in HK1b (H522, D1185) did not abolish either kinase function or osmosensibility. These results were not in agreement with those observed for HK1a [30], AHK1 [18] and other signaling pathway HK proteins, such as AHK4 involved in the cytokinin signaling pathway [49] or ETR1 involved in the ethylene signaling pathway, mainly for histidine residue [50,51]. These surprising results led us to go further to determine which one could replace the canonical amino acids for the kinase activity. Consequently, we decided to perform double and triple mutations including the “pseudo canonical” residues. As observed, each double or triple mutant failed to restore osmosensing activity in stressed conditions. However, a slight discrepancy was observed between aspartate and histidine mutants. A weak growth was noticed on growth rescue medium for aspartate mutants when not diluted but not for histidine ones. It is interesting to note that such a different behavior between canonical residues was already observed for the cytokinin receptor AHK4 in *Arabidopsis* [52]. In this HK, a histidine mutant was unable to complement a yeast strain deleted for its osmosensor Sln1p but the aspartate mutant succeeded.

Altogether, these results highlight a very different behavior between HK1a and HK1b for kinase function and osmosensibility, even though they share more than 96% similarities. Probably some structural changes could be responsible for this different behavior. It is known that for prokaryotic HKs, subtle changes in the linker domain between the DHp domain, containing the phosphoaccepting histidine residue, and the CA domain, containing the catalytic boxes (N, G1, F, G2), could affect the protein folding and also the disposition of the phosphoaccepting histidine residue [53,54,55]. It has been shown that some modifications in the linker domain of a bacterial HK, such as a proline instead of a leucine residue, can increase by 12-fold the kinase activity [53]. In poplar HK1 proteins, only one amino acid is different between HK1a and HK1b in the linker domain, but two drastic modifications occur just downstream, between the N and G1 boxes of both proteins. HK1b differs from HK1a by the lack of the asparagine 653 and a proline residue (P678) instead of an isoleucine in HK1a (Figure S4). This kind of modification could be responsible for the better performance of histidine kinase of HK1b compared to HK1a and could explain the permissiveness towards the histidine and aspartate residues’ phosphorylation. Similarly, the double proline residues observed nearby the canonical aspartate in HK1b, but not in HK1a, could be involved in the modularity of the canonical aspartate phosphorylation. It is noteworthy that HK1a was able to homodimerize in the LexA based 2H system but HK1b was not. The differential behavior of these fusion proteins could also be indicative of such a structural difference between both HK1 proteins. Structural analysis of these domains could give response elements to this atypical behavior of HK1b. Altogether, these data could, in part, explain the better performance of HK1b in the functional complementation assay.

Our study revealed a functional divergence of poplar HK1 paralogs in kinase function and osmosensibility. These new data present a new point of view in the mechanism of this MSP where two paralog receptors could have distinct performances in the same pathway. This assumption corroborates the idea that gene duplication could be an important process in plant adaptation to abiotic stress such as drought.

## 4. Materials and Methods

### 4.1. Isolation of HK1b cDNA

After identification of a second gene model corresponding to another homologous gene of *Arabidopsis thaliana* AHK1 in Phytozome 11 (*Populus trichocarpa* v3.0), specific primers were designed according to the 5′ untranslated region (UTR) sequence just upstream of the start codon, and the 3′ UTR sequence just downstream of the stop codon. PCR was performed using *Populus deltoides* (Bartr.) Marsh x *P. nigra* L. Dorskamp roots cDNAs with Q5 DNA polymerase (New England Biolabs, Ipswich, MA, USA). This amplified DNA fragment was finally cloned into the pGEM-T Easy vector (Promega, Madison, WI, USA).

Amino acid sequences of *Populus* HK1a/b were aligned with others HK1 sequences from numerous plants by ClustalW and the alignment was represented by a phylogram constructed with the neighbour-joining method in the phylogenetic software MEGA (v 6.06) (Pennsylvania State University, State College, PA, USA).

### 4.2. Construction of HK1b and HPt Triple Mutants

Mutations in HPt1 and HPt2 were introduced by PCR directly into pGAD-HPt1 and pGAD-HPt2 clones respectively, according to manufacturer’s protocol (Phusion site-directed mutagenesis kit, Finnzymes). HPt1 was mutated in order to introduce the amino acids corresponding to HPt2 at the same position and vice versa. Mutations in HK1b were introduced by PCR with specific mutated primers into pGEM-T clones in the same conditions and the resulting *HK1b* mutated sequences were cloned into the pYX212 vector by homologous recombination (InFusion, Clontech, Moutain View, CA, USA). The mutations were introduced sequentially from wild-type clones. The correct mutated sequences were confirmed by sequencing and clones were directly transformed into yeast for testing.

### 4.3. Yeast Two-Hybrid Tests

The two-hybrid assays were performed using a LexA DNA-binding domain encoding bait vector (pBTM116 referred as pLex) and a Gal4 activation domain encoding prey vector (pGADT7, Clontech). The *HK1* Cytoplasmic Part sequence (CP, amino acids 490–1249) and Receiver Domain (RD, amino acids 1089–1249) were cloned into the pLex vector as *Eco*RI-*Sal*I fragments derived from the PCR-amplified pGEMT-HK1 clone. HPts coding sequence (CDS) were cloned into the pGAD vector. The yeast strain L40Δ (*MATa ade2-101 his3-200 leu2-3,112 trp1-901 ura3-52 LYS2::(lexA op)x4-HIS3 URA3::(lexA op)x8-lacZ gal4∆*) was used for transformations according to [56]. Co-transformed yeasts were selected onto leucine–tryptophan lacking medium (−LW) for 4 days at 30 °C and streaked onto leucine–tryptophan–histidine lacking medium (−LWH) for 4 days at 30 °C. Due to weak autoactivation of hybrid proteins, −LWH medium was supplemented with 4 to 9 mM of 3-amino-1,2,4-triazole (3AT). X-Gal assays were performed according to [57]. The blue colour was allowed to appear for 1 to 7 h at 30 °C. Interactions were tested using two different reporter genes (HIS3 and LacZ) and all interactions were tested at least twice.

For the homodimerization test, the yeast strain PJ696 (MATa gal 4∆ gal 80∆ ade2-101 his3-200 leu2-3,-112 trp1-901 ura3-52 met2::GAL7-lacZ LYS2::GAL1-HIS3 GAL2-ADE2 cyh^r^2) was used. For this 2H system, HK1a/b sequences were cloned into the pGBKT7 vector (Clontech).

### 4.4. Quantitative β-Galactosidase Assay

Yeast colonies grown onto −LW medium were suspended into Z buffer at an optical density (OD)_600_ between 0.6 and 0.8 and concentrated in a final volume of 300 µL. Cells were lysed by three cycles of liquid nitrogen/37 °C incubations and 100 µL was used for triplicate tests. Two hundred µL of *ortho*-nitrophenyl-β-d-galactopyranoside (ONPG, 4 mg/mL) into Z buffer was added to the cells and the reaction was incubated at 37 °C for 10 min (HK1-RD/HPt) or 30 min (HK1-CP/HPt), then stopped with 200 µL of 1 M Na_2_CO_3_. Three hundred µL of supernatant was transferred into a microplate for OD_420_ reading. Units of β-galactosidase were expressed as Miller units. For each interaction test, a minimum of four independent clones were used. The significant difference from the wild-type interaction was estimated with a *U* test of Mann-Whitney using the R statistical software (v 3.0.0) (R fundation for statistical computing, Vienna, Austria).

### 4.5. Western Blot Analysis of Yeast Extracts

The co-transformant yeasts were cultured in the vectors’ selective medium (–LW) overnight, diluted into the same medium, and cultured to mid-log phase at 30 °C for 7 h. Then, a cell amount corresponding to 4 OD_600_ was collected by centrifugation. The cell pellets were suspended in 30 µL of lysis buffer (80 mM Tris-HCl pH 6.8, 1 mM ethylenediaminetetraacetic acid (EDTA), 10% glycerol, 2% sodium dodecyl sulfate (SDS), 100 mM dithiothreitol (DTT), 1 mM phenylmethanesulfonyl fluoride (PMSF), 0.1 mg/mL bromophenol blue and yeast inhibitor proteases cocktail) and disrupted with glass beads after heating at 100 °C. Obtained cell lysates were supplemented with 70 µL of ESB buffer and heated at 100 °C for 2 more min. Clear supernatants from centrifugation were then subjected to SDS-PAGE. Proteins transferred onto polyvinylidene difluoride (PVDF) membrane were immunoblotted with anti-LexA antibody (1:2000, DNA-binding domain, Millipore, Billerica, MA, USA) or anti-Gal4 antibody (1:2000, activation domain, Sigma, Darmstadt, Germany). A secondary anti-rabbit IgG antibody conjugated to alkaline phosphatase (1:30,000, Sigma) was used and colorimetric revelation was performed with a NBT/BCIP mixture (Western blue, Promega) for 1 h.

### 4.6. Bimolecular Fluorescent Complementation (BiFC)

BiFC assays were conducted using the pSPYCE(MR) [58] and pSPYNE173 plasmids [59] which allow the expression of a protein fused to the C- or N-terminal of the split-yellow fluorescent protein (YFP) fragments, respectively. The cDNA of HK1b-CP was cloned into *Spe*I site via homologous recombination (InFusion, Clontech) in frame with the N-terminal fragment of YFP. The coding sequences of HPt2, 7 and 9 were cloned into the *Spe*I site via enzymatic restriction whereas HPt8 and 10 were cloned into the *Sal*I site via homologous recombination (InFusion, Clontech), all in frame with the C-terminal fragment of YFP.

Transient transformation of *Catharanthus roseus* cells by particle bombardment and YFP imaging were performed according to [60] with adaptation for BiFC assays [59].

### 4.7. Expression of the HK1b Gene

Cuttings of *Populus deltoides* (Bartr.) Marsh x *P. nigra* L., clone Dorskamp, were grown in hydroponic conditions according to [61] with standard medium (MS). Osmotic stress was imposed to poplars by supplementing the medium with polyethylene glycol (PEG) 6000 at 50 g/L for 10 min. Roots, stems, petioles and leaf blades in control and stressed conditions were then harvested and frozen in liquid nitrogen. Total RNAs were extracted using the NucleoSpin^®^ RNA Plant mini kit (Macherey-Nagel, Düren, Germany) according to manufacturer’s protocol, and reverse transcription was done with ProtoScript II (New England Biolabs) using one µg of RNAs. Specific *HK1b* primers were used at 0.2 µM for amplification of one µL of cDNAs by PCR (32 cycles). *Clathrin* cDNA expression was used as the amplification control (27 cycles) and PCRs were done in triplicate, using cDNAs from two biological experiments.

### 4.8. Complementation Analysis of the sln1Δ sho1Δ Deletion Mutant MH179

The full coding sequence of the *Populus* HK1b was amplified by PCR with sequence extensions homologous to the flanking sequences of the linearization site in the pYX212 vector (*Nco*I-*Xho*I) to allow constitutive expression of HK1b, with the *URA3* gene as the selection marker.

The pPD2133 plasmid expressing the Sln1p osmosensor was used as the positive control (pPD-Sln1). The yeast strain MH179 (*ura3 leu2 his3 sln1::LEU2sho1::TRP1* + pGP22, with pGP22: *GALp-PTP2/pRS413 (HIS3*, *CEN)*) was used for transformation. Yeast cells were grown on galactose-containing medium lacking uracil (SG-Ura) for the transformation control and spotted onto SD-Ura medium in absence or presence of 0.3, 0.6, 0.9 M NaCl for 4 days at 30 °C for osmosensing complementation tests.

## Supplementary Materials

Supplementary materials can be found at www.mdpi.com/1422-0067/17/12/2061/s1.
