# Supplementary material for: Functional Divergence of Poplar Histidine-Aspartate Kinase HK1 Paralogs in Response to Osmotic Stress
Source: Int J Mol Sci. 2016 Dec 8;17(12):2061. doi: 10.3390/ijms17122061 (PMC5187861; doi:10.3390/ijms17122061)
Supplement: Supplementary file 1 [file ijms-17-02061-s001.pdf]

Supplementary Materials: Functional Divergence of Poplar Histidine-Aspartate Kinase HK1 Paralogs in Response to Osmotic Stress

François Héricourt, Françoise Chefdor, Inès Djeghdir, Mélanie Larcher, Florent Lafontaine, Vincent Courdavault, Daniel Auguin, Franck Coste, Christiane Depierreux, Mirai Tanigawa, Tatsuya Maeda, Gaëlle Glévarec and Sabine Carpin

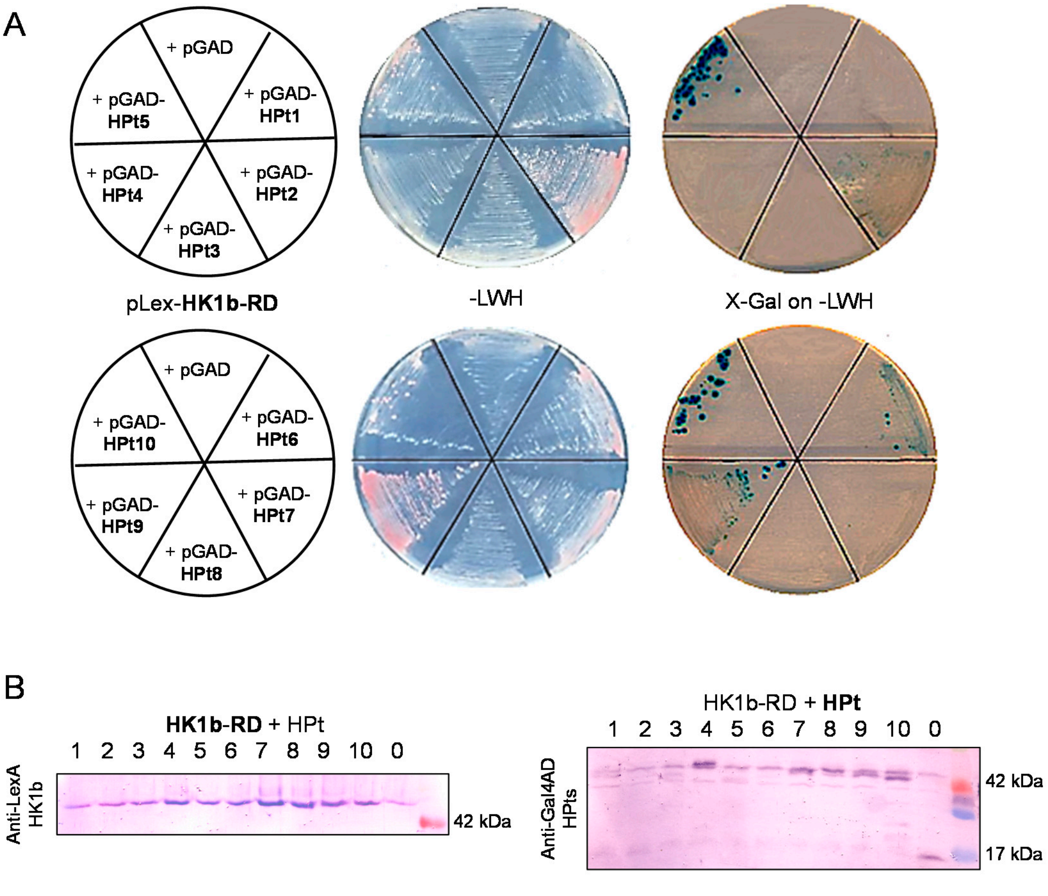

Figure S1. Interaction between HK1b and HPts. (**A**) HK1b-RD was tested with all HPts as indicated and transformed yeasts were streaked onto selective medium (-LWH) for the growth test and X-Gal test; (**B**) Fusion proteins expression is analysed by Western blot with anti-LexA and anti-Gal4AD antibodies for Lex-HK1b-RD and GAD-HPt fusion proteins immunodetection, respectively.

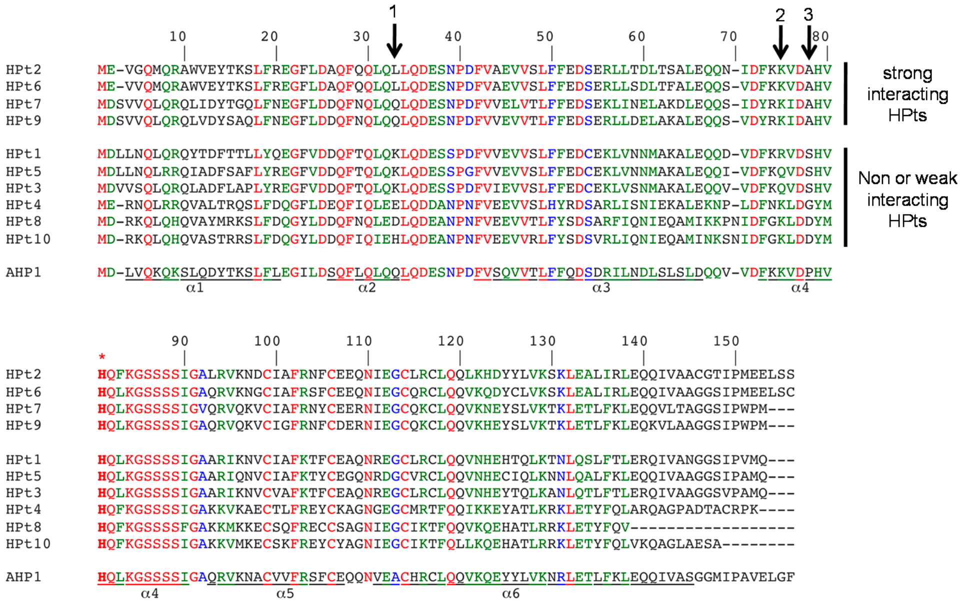

Figure S2. Alignment of HPt proteins for amino acids targeting. Amino acid sequences of all HPts were aligned and divided into two groups according to their interaction strength observed in the two-hybrid system. The sequence of AHP1 from *Arabidopsis thaliana* is shown and the helices known to interface the receiver domain in the AHK5RD/AHP1 complex structure (accession code in PDB 4EUK) are underlined. The three mutated residues are indicated by arrows and the canonical His residue acceptor of phosphorylation is shown in bold with an asterisk above. Identical amino acids are represented in red, similar amino acids in blue or green and different amino acids in black.

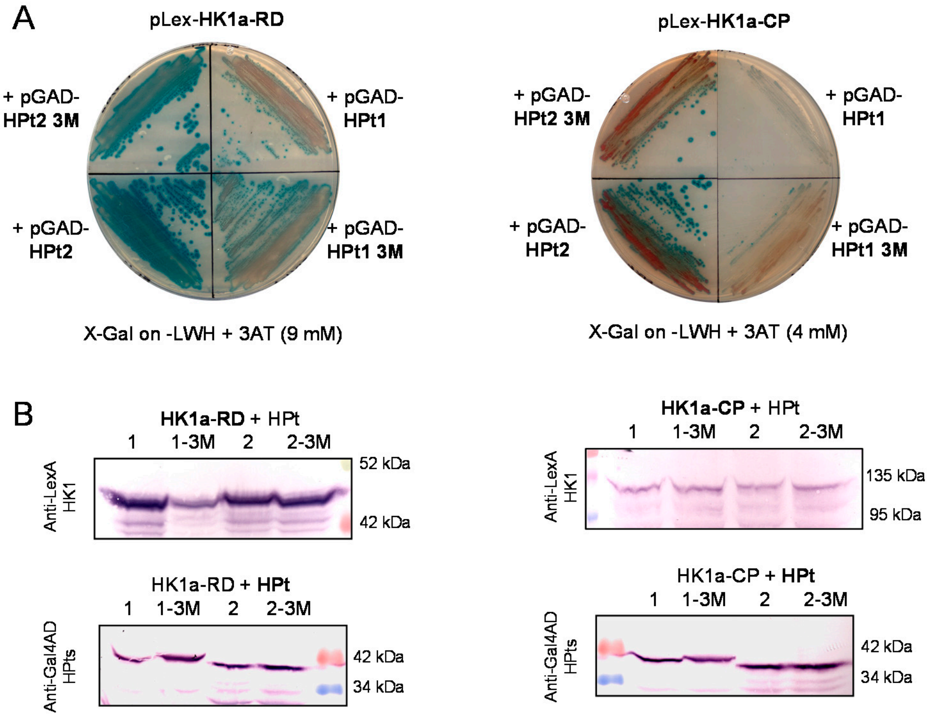

Figure S3. Interaction between HK1a/b and mutated HPt1/2. (**A**) HK1a-RD and -CP interactions with wild-type (WT) HPt1 and HPt2 and corresponding triple mutants (3M) are indicated on the X-Gal assay plates; (**B**) Fusion proteins expression is analysed by Western blot with anti-LexA and anti-Gal4AD antibodies for Lex-HK1 and GAD-HPt fusion proteins immunodetection, respectively.

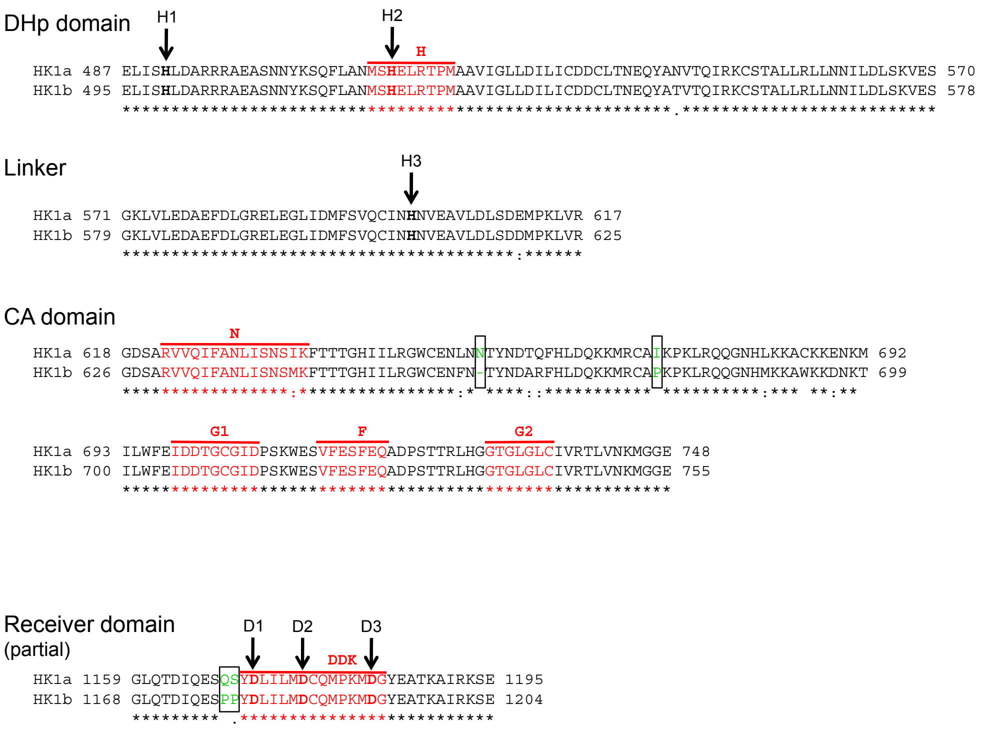

Figure S4. Alignment of poplar HK proteins. Amino acid sequences of HK1a and HK1b were aligned using ClustalW. The different motifs are represented in red and the structurally different amino acids are represented in green and boxed. The mutated residues are shown in bold and indicated by arrows. Sequence conservation is represented by (*) for amino acid identity, by (:) for strong amino acid similarity and by (.) for weak amino acid similarity. Amino acid positions are indicated by numbers on the left and right of the protein sequences.
